# Efficient Uptake of Blood-Borne BK and JC Polyomavirus-Like Particles in Endothelial Cells of Liver Sinusoids and Renal Vasa Recta

**DOI:** 10.1371/journal.pone.0111762

**Published:** 2014-11-06

**Authors:** Jaione Simon-Santamaria, Christine Hanssen Rinaldo, Piotr Kardas, Ruomei Li, Ivana Malovic, Kjetil Elvevold, Peter McCourt, Bård Smedsrød, Hans H. Hirsch, Karen Kristine Sørensen

**Affiliations:** 1 Department of Medical Biology, UiT – The Arctic University of Norway, Tromsø, Norway; 2 Department of Microbiology and Infection Control, University Hospital of North Norway, Tromsø, Norway; 3 Department of Clinical Medicine, UiT - The Arctic University of Norway, Tromsø, Norway; 4 Department of Biomedicine, University of Basel, Basel, Switzerland; 5 Divisions of Infectious Diseases and Hospital Epidemiology, University Hospital of Basel, Basel, Switzerland; University of Kansas Medical Center, United States of America

## Abstract

Liver sinusoidal endothelial cells (LSECs) are specialized scavenger cells that mediate high-capacity clearance of soluble waste macromolecules and colloid material, including blood-borne adenovirus. To explore if LSECs function as a sink for other viruses in blood, we studied the fate of virus-like particles (VLPs) of two ubiquitous human DNA viruses, BK and JC polyomavirus, in mice. Like complete virions, VLPs specifically bind to receptors and enter cells, but unlike complete virions, they cannot replicate. ^125^I-labeled VLPs were used to assess blood decay, organ-, and hepatocellular distribution of ligand, and non-labeled VLPs to examine cellular uptake by immunohisto- and -cytochemistry. BK- and JC-VLPs rapidly distributed to liver, with lesser uptake in kidney and spleen. Liver uptake was predominantly in LSECs. Blood half-life (∼1 min), and tissue distribution of JC-VLPs and two JC-VLP-mutants (L55F and S269F) that lack sialic acid binding affinity, were similar, indicating involvement of non-sialic acid receptors in cellular uptake. Liver uptake was not mediated by scavenger receptors. In spleen, the VLPs localized to the red pulp marginal zone reticuloendothelium, and in kidney to the endothelial lining of vasa recta segments, and the transitional epithelium of renal pelvis. Most VLP-positive vessels in renal medulla did not express PV-1/Meca 32, suggesting location to the non-fenestrated part of vasa recta. The endothelial cells of these vessels also efficiently endocytosed a scavenger receptor ligand, formaldehyde-denatured albumin, suggesting high endocytic activity compared to other renal endothelia. We conclude that LSECs very effectively cleared a large fraction of blood-borne BK- and JC-VLPs, indicating a central role of these cells in early removal of polyomavirus from the circulation. In addition, we report the novel finding that a subpopulation of endothelial cells in kidney, the main organ of polyomavirus persistence, showed selective and rapid uptake of VLPs, suggesting a role in viremic organ tropism.

## Introduction

The mucosal surfaces of the respiratory, oral-pharyngeal and gastrointestinal tracts are constantly exposed to a multitude of different viruses, only some of which eventually cause infection and disease [1]. Similarly to bacteria, viruses are likely to leak from the gut to the portal vein, and many of these viruses end up in the liver [2]–[8]. Some picornaviruses such as hepatitis A virus initially replicate in the intestinal mucosal surface and cause acute hepatitis when reaching the liver. Other viruses such as cytomegalovirus, Epstein-Barr virus, adenovirus, parvovirus B19, and polyomavirus cause only limited liver damage, if at all.

The liver has a critical role in pathogen detection and host defense, and contains the single largest number of macrophages, the highest concentration of natural killer cells and natural killer T cells, and the largest reticuloendothelial network in the body [9]. Of the various liver cells involved in host defense, liver sinusoidal endothelial cells (LSECs), which line the numerous sinusoids of this organ, are the least known. However, studies over the last three decades have revealed that these cells are highly active scavenger cells, removing large amounts of soluble waste macromolecules and nanoparticulate material from blood each day [10]. Thus, LSECS clearly play an important role as sentinel cells of the immune system due to: **1**) their location exposing them to 25–30% of cardiac output; **2**) their expression of several molecules involved in innate immunity such as various scavenger receptors [11]–[13]), the mannose receptor [14], [15], toll-like receptors 2–4, 7 and 9 [16]–[19], and **3**) their capacity to produce cytokines and other molecules to alert their environment [16], [19]. Furthermore, LSECs express Fc gamma-receptor IIb2 [20], [21], which mediates endocytosis of soluble immune complexes, and other receptors involved in virus uptake such as L-SIGN [22] and LSECtin [23]. Ligands eliminated from blood mainly by LSECs, and to a lesser by Kupffer cells (resident liver macrophages), include connective tissue turnover products, lysosomal enzymes [13], [15],[24]–[26]), modified plasma proteins and lipoproteins [27]–[30], soluble IgG immune complexes [31], yeast mannan [32], and adenovirus/adenoviral vectors [7],[33]. Differently to macrophages, LSECs operate essentially via clathrin-mediated endocytosis, and not by phagocytosis, and the cells are equipped with an endocytic machinery capable of super-efficient uptake and degradation of the internalized ligands [10], [34]. We have previously shown that this high scavenging activity of LSECs is conserved among all land-based vertebrates (amphibians, reptiles, birds, mammals) suggesting that this is a general mechanism for how soluble macromolecular waste material, and small colloidal particles, such as viruses, are cleared from blood [10], [35].

Cellular uptake of virus has traditionally been studied in the context of productive infection with disease-forming virus in permissive cells. Apart from the recently reported central role of LSECs in clearing blood-borne adenovirus and adenoviral vectors [7], [33], little is actually known about the role of LSECs in the uptake of virus. To explore the hypothesis that these cells act as general virus scavengers, we have studied the circulatory fate of virus-like particles (VLPs) of two non-enveloped, human DNA viruses; polyomavirus BK and JC (BKV and JCV), in mice. These viruses are approximately 40–45 nm in diameter, and are taken up in their permissive host cells via caveolin-mediated and clathrin-mediated endocytosis, respectively [36]. The VLPs represent a well-established model for endocytosis studies [37], [38]. These particles are empty virus capsids consisting of 72 capsomers of polyoma viral protein 1 (VP1), which mediate the virus binding to sialic acid receptors during infection of susceptible cells [39].

BKV and JCV infect most people early in life and establish lifelong persistent infections in the renourinary tract with asymptomatic episodes of viral reactivation and shedding in urine. While reactivation of BKV may result in severe diseases in the kidney graft and bladder of kidney- and hematopoietic stem cell transplant patients [40], JCV may lead to severe demyelinating brain disease, progressive multifocal leukoencephalopathy (PML) in immunocompromised individuals, and in patients treated with novel immunomodulatory or depleting therapies for autoimmune diseases [41]. Following respiratory or gastrointestinal transmission, BKV and JCV colonize the renourinary tract as the major site of persistence. To reach the renourinary tract, a viremic phase has been postulated, but the fate of BKV and JCV when they first appear in the circulation is currently unknown. Like most polyomavirus, BKV and JCV replication is highly specific to the human host, which hampered studies of replicative disease in animal models. However, the alpha(2,3)-linked and alpha(2,6)-linked sialic acid receptors used by BKV and JCV, respectively, are widespread in vertebrates, including mice. This encouraged us to study the fate of BK- and JC-VLPs in the blood and their cellular site of uptake in a mouse model similar to other recent studies [37], [42].

Here we present evidence for a central role of LSECs in the initial uptake of polyomavirus from blood. Despite the fact that mouse is not a permissive host for BKV and JCV, LSEC endocytic functions and receptor expression are highly similar between mammalian species, including humans [10], [11], [15], [43]–[45], arguing that human LSECs are likely to also take up these virus particles when they first appear in the circulation. In addition, we found that the BK- and JC-VLPs rapidly distributed to a distinct population of endothelial cells in kidney medulla. These endothelial cells also avidly endocytosed a scavenger receptor ligand, suggesting that they represent a subpopulation of endothelial cells with extraordinarily high endocytic activity.

## Materials and Methods

### Ethics statement

All animal experiments were approved by the Norwegian National Animal Research Authority (NARA) (approval IDs: ID-1912, ID-2775, ID-4720, ID-4894). The substances used for mouse anesthesia were either 1) a combination of tiletamine hydrochloride (Zoletil forte vet, Virbac) and xylazine (Narcoxyl vet, Intervet), or 2) a combination of fentanyl/fluanisone (Hypnorm, VetaPharma) and midazolam (Dormicum, Roche). The mice were euthanized in 100% CO_2_, while anesthetized as described above.

### Animals

Wild-type C57BL/6 male mice were obtained from Charles River laboratory (France). Mannose-receptor knock-out (MR^-/-^) C57BL/6 mice [46] were kindly provided by Professor Michel Nussenzweig, The Rockefeller University, New York, NY. The MR^-/-^ status was tested by polymerase chain reaction [46]. The animals were housed in rooms specially designed for mice, with 12 h/12 h day-night cycle, and free access to water and food (standard chow, Scanbur BK, Nittedal, Norway).

### Chemicals and reagents

Liberase TM (collagenase) was from Roche Applied Science (Oslo, Norway), Iodogen™ from Pierce Chemicals (Rockford, IL), and carrier-free Na125I from PerkinElmer Norge AS (Oslo, Norway). Bovine serum albumin was from MP Biomedicals (Solon, OH), bovine collagen type I (Vitrogen 100) from Cohesion Technologies (Palo Alto, CA), and Percoll™ and PD-10 columns (Sephadex G-25) from GE Healthcare (Uppsala, Sweden). Roswell Park Memorial Institute (RPMI) 1640 cell culture medium (supplemented with 20 mM sodium bicarbonate, 0.006% penicillin, and 0.01% streptomycin) was from Sigma-Aldrich (St Louis, MO), and Falcon cell culture plates from BD Biosciences (San Jose, CA). Draq5 was from Biostatus limited (Leicestershire, UK). Antibodies and suppliers are listed in Table 1.

**Table:** **Table 1.** Antibodies and lectins used in the study.

Antigen	Host	Clone	Isotype	Source	Dilution
BK-VP1	Rabbit	Antiserum	-	[53]	1∶1600
Mannose receptor (CD206)	Goat	Polyclonal	IgG	R&D Systems, AF2534	1 µg/ml
F4/80	Rat	Cl:A31	IgG2b	AbD Serotec, MCA497G	10 µg/ml
CD31	Rat	MEC13.3	IgG2a	Biolegend, 102502	5 µg/ml
CD68	Rat	FA-11	IgG2a	Abcam, ab53444	10 µg/ml
CD163	Rat	ED2	IgG1	AbD Serotec, MCA342GA	10 µg/ml
Meca 32	Rat	-	IgG2a	Antibodies online ABIN298904	10 µg/ml
Biotinylated *Sambucus nigra* agglutinin	-	-	-	Vector Laboratories B-1305	10 µg/ml
Biotinylated *Maackia amurensis II* hemagglutinin	-	-	-	Vector Laboratories B-1265	10 µg/ml

Secondary antibodies: Alexa488-, Alexa546-, or Alexa555- anti-rat, anti-rabbit or anti-goat IgG (H+L), raised in donkey, goat, or rabbit, all diluted 1∶500 (Life Technologies; Catalog No: A11006, A11008, A11055, A11081, A11010, A21431, A21434, A21428).

Positive lectin labeling was visualized by Alexa555-streptavidin, diluted 1∶200 (Life Technologies; Catalog No S-21381).

### Cells

Hepatic non-parenchymal liver cells (NPCs) were prepared by collagenase (Liberase) perfusion of mouse livers, followed by low-speed differential centrifugation to remove hepatocytes, and density sedimentation of the NPCs on 25%–45% Percoll gradients to collect LSEC and Kupffer cells, as described in [47]. Purified LSECs (>90% purity) were obtained after a panning step (8 min, 37°C), to remove Kupffer cells. Primary cell cultures were established on glass cover slides (mixed LSEC and Kupffer cells), and in Falcon plates (LSECs), pre-coated with 2.7 µg/ml collagen type I and maintained in serum-free RPMI-1640 medium at 37°C at 5% CO_2_. The cultures were washed 40 min after seeding, and allowed to continue spreading for approximately 1 h before use. Seeding of 3×10^5^ LSEC/cm^2^ gave confluent monolayers, with approximately 1.5×10^5^ cells per cm^2^.

### Virus-like particles (VLPs)

**Figure 1:**
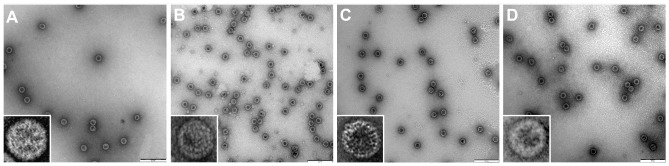
Electron microscopy of BK- and JC-virus like particles (VLPs). The figure show transmission electron micrographs of negatively stained BK- and JC-VLPs constructed from BK-VP1 or JC-VP1 proteins: A) BK-VP1 VLPs (Dunlop strain); B) JC-VP1-Mad-1 VLPs; C) JC-VP1-L55F VLPs; D) JC-VP1-S269F VLPs. Scale bars: 200 nm.

BK- and JC-VLPs were prepared as described previously [48]. Briefly, BK- and JC-VLPs were isolated from Sf9 insect cells infected with recombinant baculovirus encoding Dunlop BK-VP1 or Mad-1 JC-VP1 capsid protein. Recombinant baculoviruses containing PML variants of JC VP1 sequences L55F or S269F were obtained by site specific mutagenesis PCR using the following primers: L55F-Fwd (5′-ACC CAG ATG AGC ATT TTA GGG GTT TTA G-3′) and L55F-Rev (5′-CTA AAA CCC CTA AAA TGC TCA TCT GGG T-3′), S269F-Fwd (5′-TAA CAG ATC TGG TTT CCA GCA GTG GAG A-3′) and S269F-Rev (5′-TCT CCA CTG CTG GAA ACC AGA TCT GTT A-3′), respectively. Infected Sf9 cells were disrupted by sonication and glass mortar and pestle treatment. VLPs were purified from cellular lysate by CsCl gradient and stored in buffer consisting of 150 mM NaCl, 10 mM Tris-HCl pH 7.4 and 1.0 mM CaCl_2_. The 3-dimensional structure and integrity of VLPs was confirmed by transmission electron microscopy (**Figure 1**). In short, JC-VLPs or BK-VLPs in 0.9% NaCl were placed on discharged pallodium coated mesh grids for 2 min; then washed twice with double distilled water, and once with freshly made 2% uranyl acetate. Another drop of 2% uranyl acetate was added for 10 sec. The grids were gently washed on drops of double distilled water and air dried before electron microscopy. The images were recorded in a FEI CM100 TEM transmission electron microscope (FEI, OR, USA), operating at 80 kV, and equipped with Veleta digital CCD camera (Olympus, Germany).

### Ligand labeling procedures

VLPs in phosphate buffered saline (PBS) were labeled with carrier-free Na^125^I, using Iodogen as described by the manufacturer (Pierce Chemicals), and separated from unbound ^125^I on a PD-10 column. The resulting specific radioactivity was approximately 4−7×10^6^ cpm per µg protein. ^125^I-VLPs for injections were mixed with 0.9% NaCl to a final concentration of approximately 0.5 µg protein/100 µl ( =  dose injected per mouse).

Fluorescein isothiocyanate-labeled formaldehyde-denatured bovine serum albumin (FITC-FSA) was prepared by incubating FSA and FITC in sodium carbonate buffer (0.5 mol/L, pH 9.5) at a protein-dye ratio of 4∶1 at 4°C overnight, and dialyzed against PBS.

### Blood clearance and organ distribution of radiolabeled VLPs

^125^I-BK-VLP, ^125^I-JC-VLP, ^125^I-JC-VLP_L55F_, or ^125^I-JC-VLP_S269F_ (approximately 0.5 µg protein) were injected into the tail vein of anaesthetized mice (n = 3–5 per group). Immediately thereafter blood samples (5 µl) were collected over short time intervals from the tail tip, mixed with 0.3 ml of 13 mM citric acid, after which 0.3 ml 2% bovine serum albumin in water and 0.6 mL of 20% (wt/vol) trichloroacetic acid were added to the samples to precipitate non-degraded protein. Acid-soluble radioactivity represented degraded ligand [25]. To test if the higher doses of VLPs injected for immunohistochemistry would influence blood clearance and organ distribution, non-labeled JC-VLPs (15 µg  =  dose used for immunohistochemistry) were injected simultaneously with ^125^I-JC-VLPs (0.5 µg). This did not change the blood clearance or organ distribution pattern of the radiolabeled ligand. In some animals FSA (0.8 mg protein in100 µl 0.9% NaCl) was injected immediately before the administration of radiolabeled ligand to check for inhibition of blood clearance.

At the end of the monitoring period (10 min or 60 min) the anaesthetized animals were euthanized with 100% CO_2_. The abdomens and thoraces were opened, and the animals were perfused via the left cardiac ventricle (outlet via the right atrium) with PBS to remove free tracer from the vasculature before the organs were excised and analyzed for radioactivity. The amount of tracer recovered was considered the sum of radioactivity of individual organs and carcass without organs.

### Hepatocellular distribution of ^125^I-VLPs

^125^I-BK-VLP, ^125^I-JC-VLP, ^125^I-JC-VLP_L55F_, or ^125^I-JC-VLP_S269F_ (approximately 0.5 µg protein) was injected into the tail vein of anaesthetized mice (n = 3 per group). Ten minutes after the injection the animals were euthanized with 100% CO_2_ and liver cells isolated by collagenase perfusion of liver via the portal vein, before separating the dispersed liver cells into hepatocytes and NPCs [47]. All steps during cell isolation and separation, except the liver perfusion step, were carried out on ice/below 4°C. Radioactivity in the hepatocyte and NPC fractions was measured in a Packard Gamma Counter. Cell numbers were assessed by cell counting in a hemocytometer.

### Immunohistochemistry and lectin histochemistry

Non-labeled BK-VLPs or JC-VLPs (13–18 µg protein per mouse) was injected into the tail vein of anaesthetized mice (n = 3–5 per ligand). The animals were euthanized after 7–15 min, then perfused with PBS through the left cardiac ventricle to remove blood and thereafter with 4% paraformaldehyde (PFA) in PBS with 0.02 M sucrose. The perfusion fixed tissues were dehydrated, paraffin embedded and sectioned. In some animals, FITC-FSA (50 µg protein per mouse) was injected into the tail vein 5 min after injection of 10 µg JC-VLP, JC-VLP_L55F_, or JC-VLP_S269F_, and the mice euthanized 10 min thereafter, before perfusion fixation and tissue preparation for immunohistochemistry as described above. Antigen retrieval was achieved by 30 min microwaving in citrate buffer, pH 6. Blocking buffer was 5% goat serum or 1% BSA in TBS and 0.05 M Tween 20, pH 8.4. Sections were incubated overnight (37°C) with primary antibodies (**Table 1**) or non-immune IgG diluted in blocking buffer, washed and incubated with secondary antibody in blocking buffer for 1 h at room temperature. Nuclear stain was Draq5 (diluted 1∶1000 in PBS). Sections were analysed in a Zeiss LSM 510 confocal laser scanning microscope (Carl Zeiss, Germany).

#### Lectin histochemistry on tissue sections

Biotinylated lectins, *Sambuccus nigra* agglutinin (SNA), and *Maackia amurensis* hemagglutinin (MAH), were purchased from Vector Laboratories (Vector Laboratories Inc, CA, USA), and Alexa555-streptavidin from Life Technologies (**Table 1**). SNA recognizes alpha(2,6)-linked sialic acids, and MAH recognizes alpha(2,3)-linked sialic acids [49]. Lectin labeling of paraffin tissue sections of liver, kidney and spleen were done according to the instructions from Vector Laboratories.

### Endocytosis experiments in isolated liver cells

#### Confocal microscopy

Primary NPC cultures, enriched in LSECs with approximately 10–20% Kupffer cells, were established on collagen coated glass cover slides. Since LSECs rapidly lose their *in vivo* uptake functions after isolation [50] endocytosis experiments were performed under serum-free conditions within 4 h of plating. Antibody markers for LSECs and Kupffer cells are listed in **Table 1**. The cells were incubated with BK-VLP, JC-VLP, or BKV for 30 min-1 h, washed, fixed in 4% PFA in PBS/0.02 M sucrose for 15 min, immune labeled, and subjected to confocal laser scanning microscopy.

#### Immune EM

Purified LSECs (approximately 1.5 million cells in 8 cm^2^ Falcon wells) were incubated with 2.06E+10 genomic equivalents of CsCl gradient purified BKV in 1 ml serum-free RPMI 1640 medium for 2 h, washed in PBS and fixed in 4% formaldehyde in 200 mM Hepes buffer, pH 7.5, overnight, embedded in gelatin and 2.3 M sucrose, and snap frozen in liquid nitrogen. Immune labeling of ultrathin cryosections was performed as described [51]. Positive BK-VP1 labeling was detected by protein A-conjugated 10 nm gold complexes. The dried sections were examined in a JOEL JEM 1010 transmission electron microscope (JOEL, Tokyo, Japan) operating at 80 kV. Control experiments were included in parallel by omission of the primary antibody.

### Statistics and pharmacokinetic analyses

The statistical calculations (Mann-Whitney U test, ANOVA two-way analysis of variance) were performed with GraphPad Prism (GraphPad Software Inc. San Diego, CA). Clearance kinetics were analyzed as described [52].

## Results

### Removal of blood-borne BK- and JC-virus like particles (VLPs) was rapid, with JC-VLPs being cleared more efficiently than BK-VLPs

**Figure 2:**
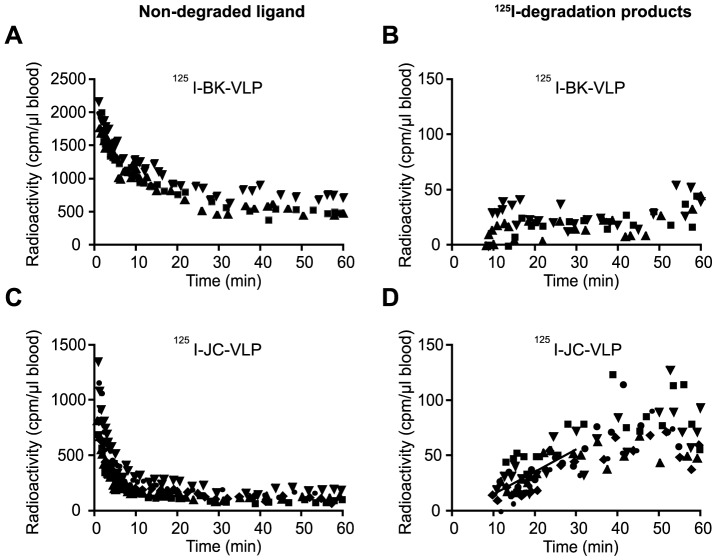
Blood clearance and subsequent in vivo degradation of BK- and JC-virus like particles (VLPs). Mice were injected intravenously with approximately 0.5 µg of ^125^I-BK-VLPs (n = 3; A, B), or ^125^I-JC-VLPs (n = 5; C, D), and blood samples taken at the indicated time points and analyzed for ^125^I-labeled degradation products and intact ligand [15]. Panels A and C show decrease of intact ligand in blood as a function of time. Panels B and D show increase in degradation products released into the blood with time. Results are given as cpm per µl blood. Different symbols refer to separate animals. cpm, counts per minute. The slope of line drawn in panel D represents the average rate of release of degradation products in the 10–30 min period after ligand injection where this release followed approximate first order kinetics.

BK-VLPs and JC-VLPs were radiolabeled and injected via the tail vein. The rate of blood clearance of ^125^I-BK-VLP and ^125^I-JC-VLP is shown in **Figure 2A, C**. Semi-logarithmic decay plots revealed a biphasic pattern of elimination from blood, with an initial rapid t_1/2_ α of 1–2 min for both ligands (**Table 2**). During this early phase the uptake from blood of ^125^I-BK-VLP and ^125^I-JC-VLP were 39% and 76%, respectively, revealing a considerably more effective initial blood clearance of JC-VLP than BK-VLP. After 60 min approximately 30% of the BK-VLP associated radioactivity was still in the circulation (**Figure 2A**), suggesting that BK-VLPs may interact with some unknown blood factor(s) protecting them from being taken up. In contrast, only 10% of JC-VLP associated radioactivity was left in blood after 30 min (**Figure 2C**).

**Table:** **Table 2.** Rate of blood clearance of BK- or JC virus-like particles (VLPs) in mouse.

Ligand	t_1/2_ α (min)*	t_1/2_ β (min)*	Fraction (%) eliminated in α phase	Fraction (%) eliminated in β phase
^125^I-BK-VLP	2.06±0.90	42.85±9.51	39.18±6.30	60.82±6.30
^125^I-JC-VLP	1.17±0.11	46.08±37.20	75.70±3.84	24.30±3.84
^125^I-JC-VLP_L55F_	1.28±0.26	25.16±12.76	79.21±2.75	20.80±2.75
^125^I-JC-VLP_S269F_	1.06±0.30	41.81±7.62	70.92±11.60	29.08±11.60
^125^I-BK-VLP+FSA	0.91±0.71	33.5±7.69	56.17±26.73	43.89±26.73
^125^I-JC-VLP+FSA	1.04±0.11	31.13±8.50	85.62±2.78	14.37±2.78

N = 3 in all groups except ^125^I-JC-VLP where n = 5. Results are given as mean ± SD.

*t_1/2_ α and t_1/2_ β were calculated as described in [52].

Intracellular degradation of ^125^I-labeled proteins normally generates ^125^I-tagged low molecular mass degradation products that are released from the cells, and can be measured in blood following trichloroacetic precipitation of intact proteins [25]. Acid soluble ^125^I-degradation products appeared in blood 10 min after injection of the radiolabeled VLPs (**Figure 2B, D**). In agreement with the more efficient blood clearance of JC-VLPs, more degradation products were released to blood after injection of ^125^I-JC-VLPs than after ^125^I-BK-VLP injection in the 60 min monitoring period.

### BK- and JC-VLPs distributed to liver, with a minor uptake in spleen and kidney

**Figure 3:**
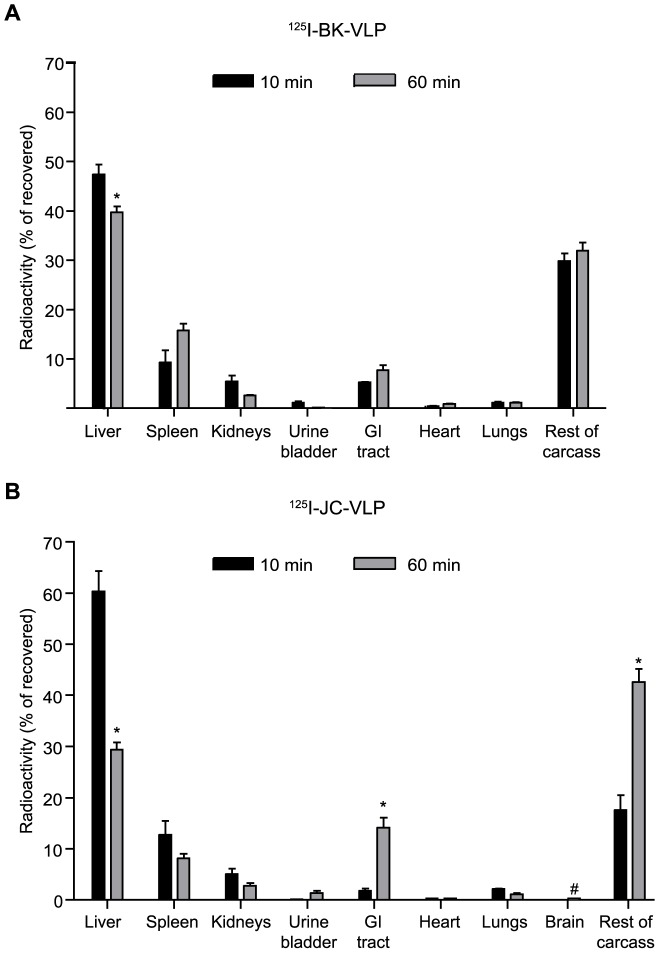
Anatomical distribution of ^125^I-labelled BK- and JC-VLPs. Mice were injected intravenously with ^125^I-labelled BK-VLPs (A) or JC-VLPs (B), euthanized after 10 min (black bars; n = 3) or 60 min (grey bars; n = 3 in A; n = 5 in B), and organs and tissues analyzed for radioactivity. The mice used in the circulatory half-life study (Figure 2) were included in this anatomical distribution study (60 min values). Total recovered radioactivity in all tissues at the given time points was taken as 100%. Error bars represent SEM. *Statistically significant difference (p<0.01) between tissue radioactivity at 10 min versus 60 min. # Brain radioactivity was measured at 60 min, only. GI tract, gastrointestinal tract (stomach and intestines including mesenterium).

To minimize the escape of label from the site of uptake due to intracellular ligand degradation, organ distribution of radiolabeled ligands was measured already 10 min after injection. At this point, the majority of the ^125^I-BK-VLP (48%) and ^125^I-JC-VLP (60%) associated radioactivity was found in liver, whereas spleen and kidneys contained 9% and 5% of the ^125^I-BK-VLPs, and 12% and 5% of the ^125^I-JC-VLPs, respectively (**Figure 3**).

To investigate the kinetics of organ distribution the VLPs following administration, the liver-associated radioactivity was followed from 10 to 60 min after injection. For ^125^I-BK-VLP, liver-associated radioactivity decreased only slightly (**Figure 3A**), which may reflect that cellular uptake and degradation overlap due to the slower blood clearance of BK-VLP. In contrast, for ^125^I-JC-VLP, the liver-associated radioactivity decreased from 60% to 30% of total recovered radioactivity during the same time period (**Figure 3B**), indicating effective hepatocellular degradation of JC-VLP. A corresponding increase of radioactivity in the gastrointestinal tract (including mesenterium and lymph nodes) and the rest of carcass in the same period was judged to be caused by redistribution of degradation products, which is commonly noted following administration of ^125^I-labeled ligands [26].

To measure the relative contribution of different liver cells in the VLP uptake mice injected intravenously with ^125^I-labelled BK- or JC-VLPs were sacrificed after 10 min for liver cell isolation and separation into hepatocytes and NPCs, the latter consisting mainly of LSECs and some Kupffer cells, as described in Materials and Methods. The liver radioactivity was mainly associated with the NPC fraction (**Table 3**).

**Table:** **Table 3.** Liver uptake of BK- and JC-VLPs from blood takes place in NPCs.

	Ratio of radioactivity in NPCs versus hepatocytes after injection of ^125^I-BK-VLPs		Ratio of radioactivity in NPCs versus hepatocytes after injection of ^125^I-JC-VLPs
Mouse 1	6.5: 1	Mouse 1	22.1: 1
Mouse 2	6.3: 1	Mouse 2	29.8: 1
Mouse 3	26.5: 1	Mouse 3	22.3: 1
Average	13.1: 1	Average	24.8: 1

The mice were injected intravenously with ^125^I-labelled VLPs, approximately 0.5 µg per animal, and euthanized after 10 min. Liver cells were then isolated by *in situ* perfusion with collagenase, and separated by low-speed differential centrifugation and subsequent density centrifugation on a Percoll gradient [47]. Radioactivity (counts per minute) was measured in the purified hepatocyte fraction, and in the NPC fraction enriched in LSECs and Kupffer cells.

### The liver uptake of BK- and JC-VLPs took place mainly in the sinusoidal endothelium

To identify the cellular distribution of VLPs in liver, non-labeled BK-VLPs or JC-VLPs were injected intravenously, and organs harvested after 7–15 min, fixed, paraffin embedded, and processed for immunohistochemistry using a rabbit antiserum against BK-VP1 [53] (**Table 1**) that cross-reacts with JC-VP1.

**Figure 4:**
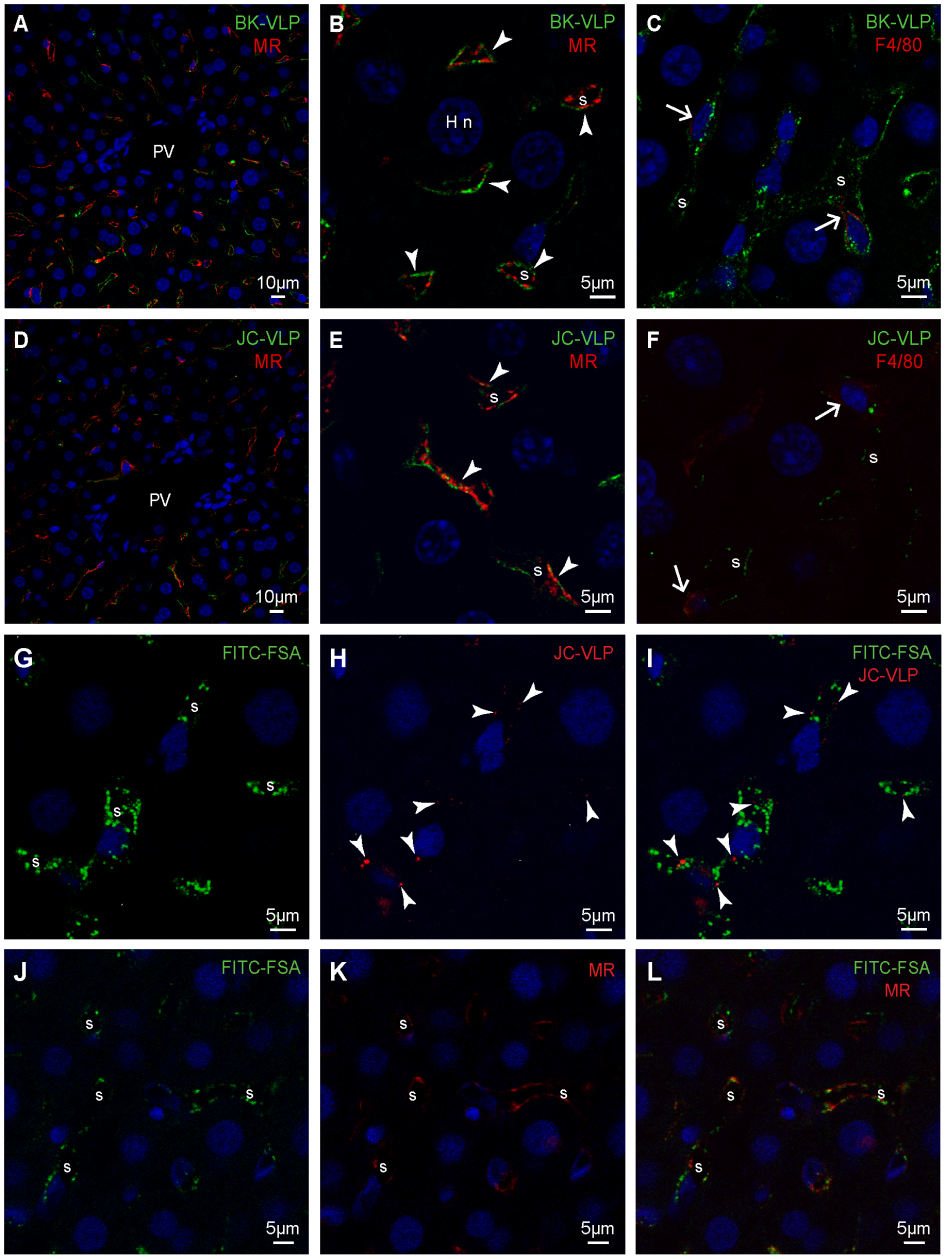
Liver cell distribution of BK- and JC-VLPs after uptake from blood. Panels A–F: Mouse livers were perfusion fixed 15 min after intravenous injection of BK-VLPs (A–C), or JC-VLPs (D–F). Paraffin embedded tissue sections were double immune labeled using a rabbit antiserum against BK-VP1 [53], and antibodies against the mannose receptor (MR), which is an LSEC marker in mouse liver [26], or F4/80 (Kupffer cell marker). Antibodies are listed in Table 1. The VP1 staining (green), indicative of BK-/JC-VLP uptake, showed a typical LSEC pattern (A, D, overview; B, E, close ups), as evidenced by similar localization of green VP1 and red MR staining along the sinusoids (arrowheads in B, E; overlap of red and green fluorescence is shown in yellow). Some BK- and JC-VLP uptake was also observed in F4/80 positive (red) Kupffer cells (arrows in C, F). PV, portal vein; S, sinusoid; Hn, nucleus of hepatocyte. Panels G–L: In G–L, FITC-labeled formaldehyde-denatured serum albumin (FITC-FSA) was injected into the tail vein 5 min after intravenous injection of JC-VLPs to functionally label the endothelium of the liver sinusoids [54], [62]. Ten min thereafter the animals were euthanized, tissues perfusion fixed, paraffin embedded and prepared for immune histochemistry. Panels G-I show the distribution of FITC-FSA (green) and JC-VLP (red, arrowheads) in liver sinusoids (s). Panels J–L show similar distribution of FITC-FSA (green) and MR expression (red) in the sinusoids, s.

In liver, BK- and JC-VLP staining was observed along the hepatic sinusoids, colocalizing with cells expressing the mannose receptor (LSEC marker) (**Figure 4A, B, D, E** **; Figure S1**), or in cells that had endocytosed the scavenger receptor ligand FITC-FSA, a functional LSEC marker [30], [54] (**Figure 4G–I**). In C57BL/6 mouse liver, the mannose receptor is expressed in LSECs but not in Kupffer cells [26]. Some uptake of BK- and JC-VLPs was also seen in F4/80 positive Kupffer cells (**Figure 4C, F**), whereas no BK- or JC-VLP positive staining was observed in hepatocytes, endothelial cells of portal venules or arteries, bile ducts or other liver structures.

**Figure 5:**
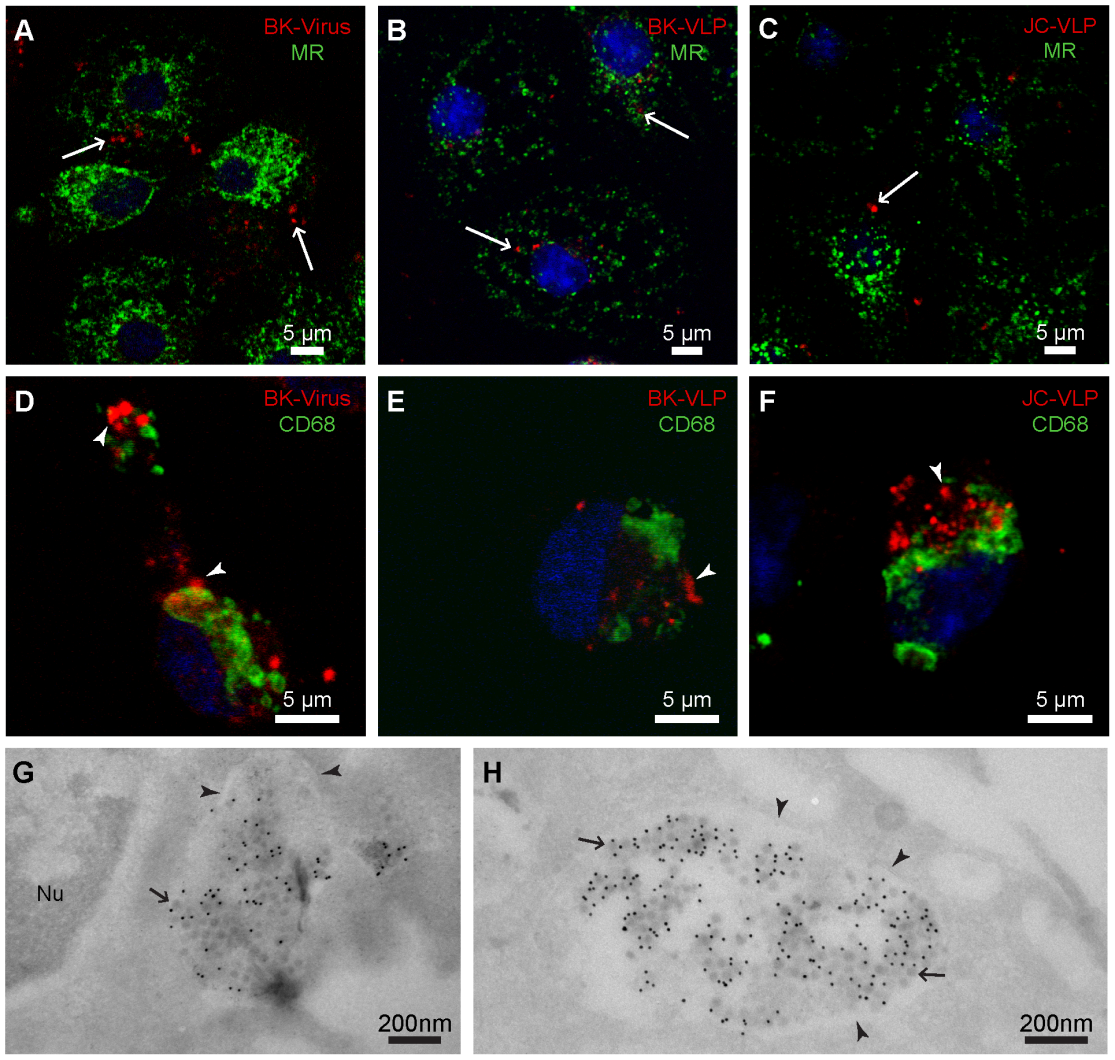
In vitro endocytosis of BK- and JC-VLPs, and BK virions in liver sinusoidal cells. Freshly isolated non-parenchymal liver cells (mixed LSEC and Kupffer cell cultures) were incubated at 37°C for 30 min with BK virions (A, D), or for 1 h with 10 µg/ml BK-VLPs (B, E), or JC-VLPs (C, F), fixed, and double immune labeled with a rabbit anti-BK-VP1 antiserum (red fluorescence) and antibodies against the mannose receptor (MR; LSEC marker; shown in green), or CD68 (Kupffer cell marker; green). Antibodies are listed in Table 1. Uptake of BK virions and BK-/JC-VLPs was observed both in isolated LSECs (arrows, A–C) and Kupffer cells (arrow heads, D–F). Panels G–H: Cryo-immune electron microscopy of BK virion uptake in LSEC membrane-bound vesicles. Purified LSEC cultures were incubated with BK virions for 2 h at 37°C. Positive BK-VP1 staining was visualized by protein-A-10 nm gold particles (black dots). Arrowheads points to vesicular membranes, and arrows to virus particles. Nu, nucleus.

To characterize in greater detail the cells involved in the hepatic uptake of polyomavirus, freshly isolated liver NPCs were incubated with infectious BKV, non-labeled BK-VLPs, or JC-VLPs for various time periods (30 min–1 h), immune labeled and subjected to confocal microscopy (**Figure 5A–F**). Both LSECs and Kupffer cells took up BK- and JC-VLPs, and BKV *in vitro*. Results were confirmed by double labeling with anti-BK-VP1 antiserum and antibodies against the mannose receptor (LSEC marker in liver [26]; **Figure 5A–C** **; Figure S2**) or CD68 (Kupffer cell marker; **Figure 5D–F**). Cryo-immune electron microscopy of purified LSEC cultures incubated with infectious BKV for 2 h showed virus accumulation in membrane-bound compartments (**Figure 5G, H**).

### Liver uptake of blood-borne VLPs was not dependent on sialic acids

**Figure 6:**
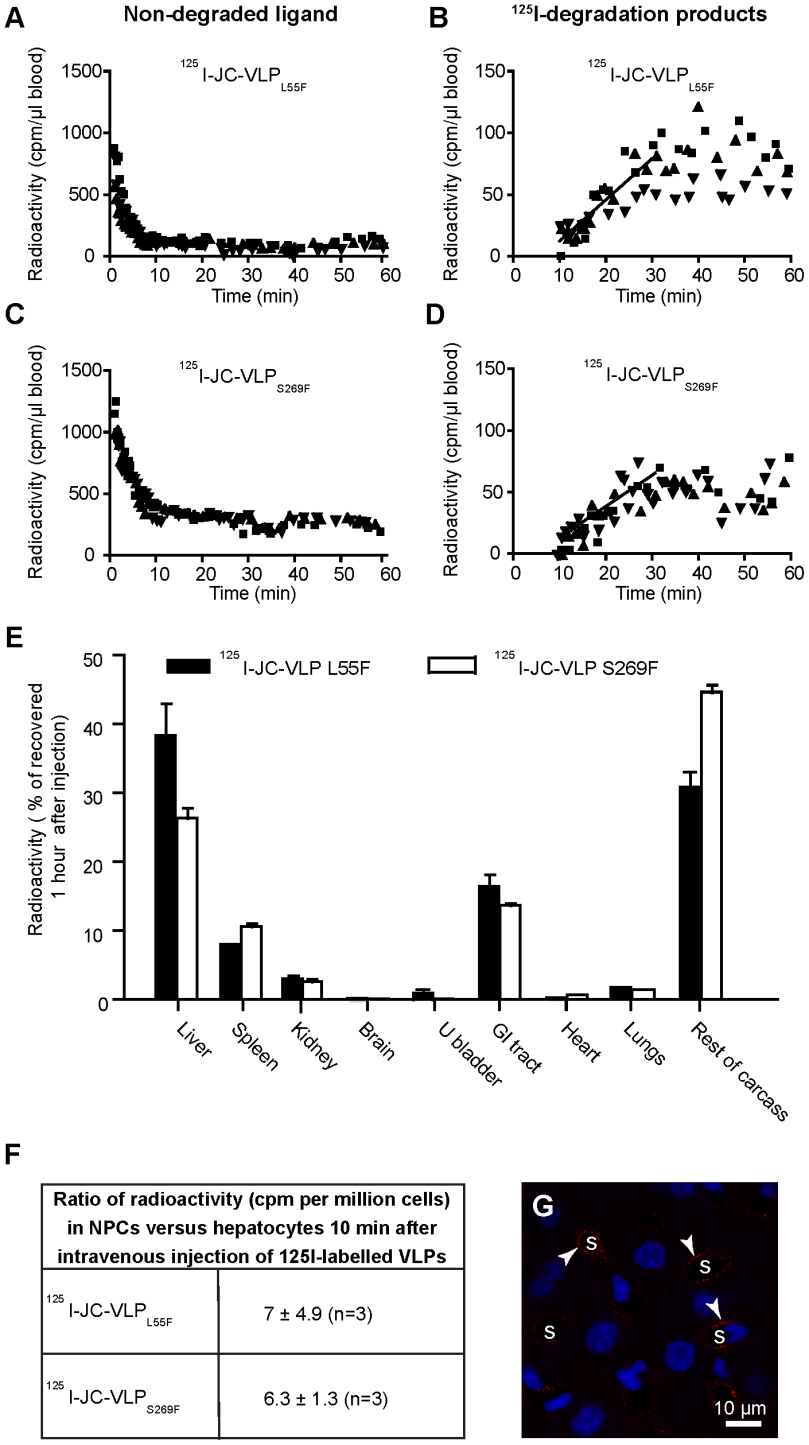
Blood clearance and organ distribution of mutant JC-VLPs. Mice were injected intravenously with ^125^I-JC-VLP_L55F_ (n = 3, A–B), or ^125^I-JC-VLP_S269F_ (n = 3, C–D), and blood samples taken at the indicated time points and analyzed for ^125^I-labeled degradation products and intact ligand [15]. Panels A and C show the decrease of intact ligand in blood as a function of time. Panels B and D show the increase in degradation products released into the blood with time. Different symbols refer to separate animals, and cpm to counts per minute. The slopes of the lines drawn in B and D represent the average release rate of degradation products in the 10–30 min period after ligand injection where this release followed approximate first order kinetics. E) Tissue distribution 60 min post injection. The animals in A–D were sacrificed after 60 min, and organs and tissues analyzed for radioactivity. Recovered radioactivity in all tissues at this time point was taken as 100%. Error bars represent SEM. GI tract, gastrointestinal tract (stomach and intestines including mesenterium); U bladder, urine bladder. F) Hepatocellular distribution (±SD) of ^125^I-labeled JC-VLP mutants 10 min after intravenous injection; liver tissue was dispersed by collagenase perfusion, and radioactivity was measured in hepatocyte and non-parenchymal cell (NPC) fractions, respectively. G) Cellular distribution of JC-VLP_S269F_ (anti-VP1 staining) 15 min after VLP injection. Uptake of JC-VLP_S269F_ (red fluorescence; arrowheads) is seen along the sinusoids (s).

It is reported that BKV and JCV enter their natural host cells via carbohydrate receptors [55]. While BKV binds to alpha(2,3)-linked sialic acids on (for instance) gangliosides [56], JCV binds to alpha(2,6)-linked glycan lactoseries tetrasaccharide c (LSTc) [57]. To test whether blood clearance of JCV in mice is dependent on alpha(2,6)-linked sialic acids, we examined the elimination from blood of two JC-VLP variants occurring frequently in PML patients, namely JC-VLP_L55F_ and JC-VLP_S269F_ (**Figure 6A–D** **, Table 2**), which had been shown to no longer hemagglutinate red blood cells and to have lost their respective sialic acid receptor binding properties [58]. These VLPs were constructed from mutant forms of the JCV capsid protein VP1, in which one amino acid in the sialic acid binding loop of VP1 had been substituted. The data show that ^125^I-JC-VLP_L55F_ and ^125^I-JC-VLP_S269F_ were removed from the circulation in a similar manner as the JC-VLPs made from Mad-1 JC-VP1 (named here as JC-VLP) (**Table 2**). After 60 min nearly all ^125^I-JC-VLP_L55F_, and 80% of the ^125^I-JC-VLP_S269F_ were eliminated from blood (**Figure 6A, C**). Similarly to the JC-VLPs, acid soluble ^125^I-degradation products appeared in the circulation already after 10 min (**Figure 6B, D**).

Organ distribution of ^125^I-JC-VLP_L55F_ and ^125^I-JC-VLP_S269F_ 60 min post injection (**Figure 6E**) was similar to that of JC-VLP (**Figure 3B**), with the highest radioactivity measured in liver. Notably, radioactivity in the brain was negligible. Hepatocellular distribution studies, monitoring radioactivity in hepatocytes and NPCs separated10 min after injection of radiolabeled ligands, showed that the uptake of ^125^I-JC-VLP_L55F_ and ^125^I-JC-VLP_S269F_ was predominantly in the NPC fraction (**Figure 6F**). Immunohistochemistry revealed an LSEC-like pattern of distribution along the sinusoids (**Figure 6G**), as with JC-VLP (**Figure 4**). Of note, lectin histochemistry for SNA and MAH revealed that all hepatic endothelia expressed alpha(2,6)-linked-sialic acids and alpha(2,3)-linked sialic acids (**Figure S3**), whereas BK-VLPs and JC-VLPs (Mad-1 and mutants) distributed only to sinusoids (**Figures 4** **, and** **6**), suggesting that these sialic acids are not essential for liver uptake.

### The scavenger receptor ligand formaldehyde-denatured serum albumin (FSA) reduced the rate of intracellular degradation of JC-VLP, but did not interfere with VLP uptake in liver

LSECs mediate their effective blood clearance of most known ligands via a limited set of endocytosis receptors of broad ligand specificity [10]. To study if LSEC scavenger receptors were involved in the uptake of circulating BK-VLPs or JC-VLPs, a high dose of FSA (0.8 mg/mouse) was injected immediately prior to the injection of ^125^I-labeled VLPs. FSA is a soluble scavenger receptor ligand and is reported to distribute mainly to the LSEC after intravenous administration [59]. No effect was observed on the blood clearance kinetics of ^125^I-BK-VLP (**Table 2**) or ^125^I-JC-VLP (**Figure S4,** **Table 2**). However, the release of ^125^I-JC-VLP degradation products to the blood (**Figure S4-B**) was slightly reduced by simultaneous FSA injection compared to injection of only ^125^I-JC-VLP (**Figure 2D**). This resulted in significantly more liver-associated radioactivity after 60 min (**Figure S4-C**) compared to injection of ^125^I-JC-VLP alone (**Figure 3B**), suggesting that metabolic turnover, but not uptake was rate limiting following targeting of the same cell by FSA and JC-VLP.

To investigate if the clearance of the VLPs was mediated by the mannose receptor, a C-type lectin with broad ligand binding properties and highly expressed in mouse, rat and human LSECs [15], [26], [43], mannose receptor deficient mice [46] were injected with ^125^I-JC-VLPs (**Figure S5**). These mice showed similar clearance and organ distribution of JC-VLPs as wild type mice (**Figures 2B** **,** **3B**), indicating that the rapid JC-VLP blood clearance was not mediated by the mannose receptor pathway.

### Extrahepatic clearance of blood-borne BK- and JC-VLPs took place in endothelial cells of kidney vasa recta segments, and in reticuloendothelial cells of spleen red pulp marginal zone

The organ distribution studies of radiolabelled ligands showed a minor, but significant uptake in kidney and spleen. This prompted us to examine the VLP uptake in these organs by immunohistochemistry.

**Figure 7:**
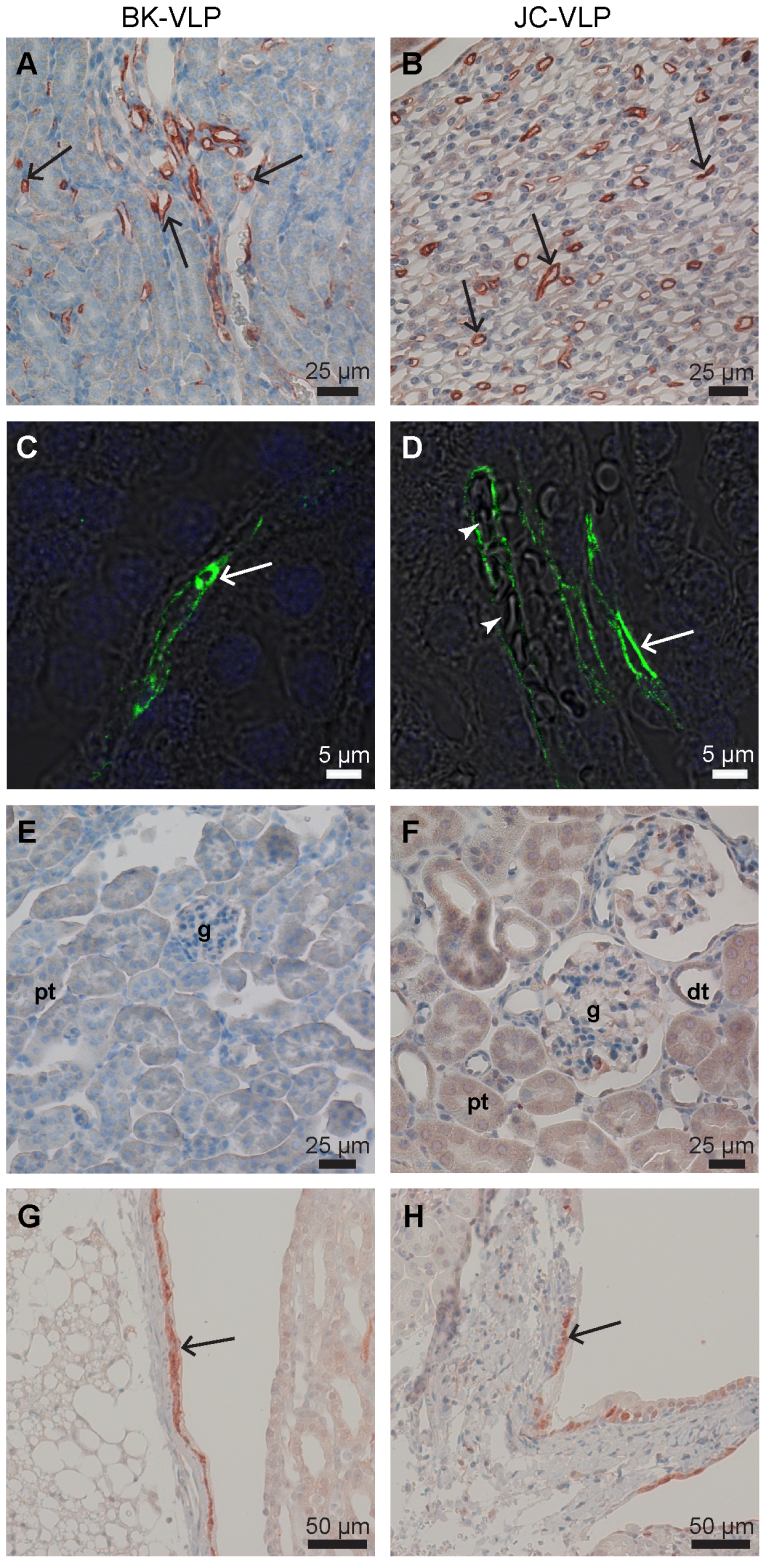
Kidney distribution of intravenously administered BK- and JC-VLPs. Mouse tissues were perfusion fixed 7 or 15 min after injection of BK-VLPs (A, C, E, G), or JC-VLPs (B, D, F, H). Paraffin sections were labeled using a rabbit anti-BK-VP1 antiserum [53]. VP1-labeling was visualized using HRP polymer/DAB reaction (brown color), or Alexa488-goat-anti-rabbit (green fluorescence). Specific uptake of VLPs was seen in the endothelial lining of microvessels in kidney medulla (arrows in A–D; with arrowheads in D pointing to blood filled medullary capillaries), whereas glomeruli (E, F), and tubular structures were negative (A–F). Epithelial cells in the transitional epithelium of renal pelvis also showed positive VP1 staining (arrows in G, H). g, glomerulus; pt, proximal tubulus; dt, distal tubulus.

**Figure 8:**
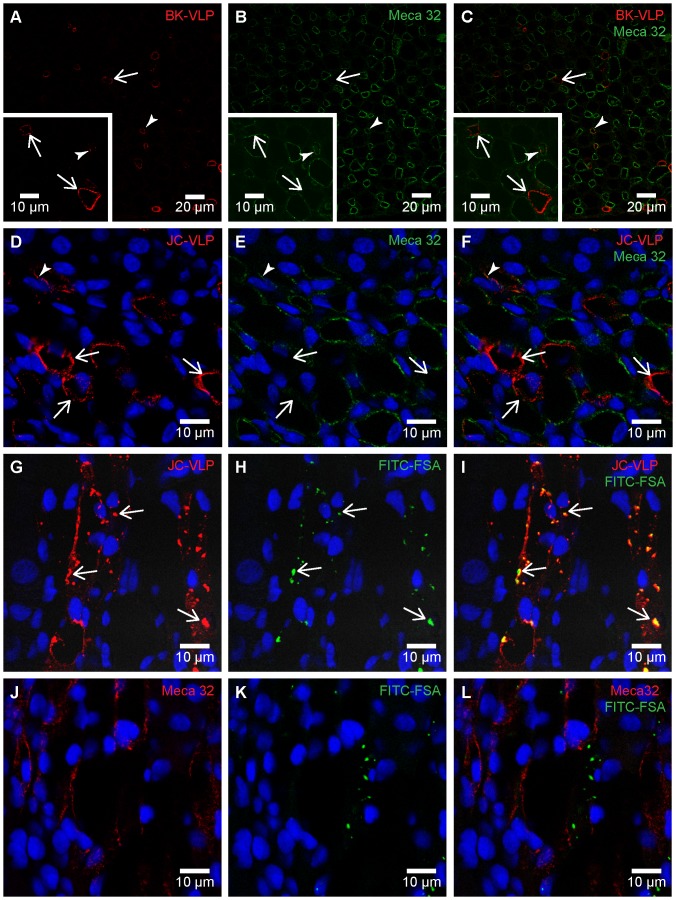
Polyoma VLP and FITC-FSA uptake in renal medullary endothelial cells. Panels A–F: Mouse tissues were perfusion fixed 7 min after intravenous injection of BK-VLP (A–C), or JC-VLP (D–F), and paraffin sections double immune labeled with anti-BK-VP1 (red) and anti-Meca 32 (green). Panels A–C: Arrows indicate endothelial uptake of BK-VLPs in Meca 32 negative vessels, and arrow heads to BK-VLP uptake in Meca 32 positive vessels. Panels D–F: Arrows indicate endothelial uptake of JC-VLPs in Meca 32 negative vessels, and arrow heads to JC-VLP uptake in Meca 32 positive vessels. Most BK-VLP or JC-VLP-positive vessels were Meca 32 negative or showed only weak Meca32 staining. Panels G–L: Tail vein injection of JC-VLPs was followed by injection of the scavenger receptor ligand FITC-FSA in the opposite tail vein and tissues perfusion fixed 15 min after the VLP administration. Paraffin sections were immune labeled with either anti-BK-VP1 (red; G–I) or anti-Meca 32 (red; J–L). G–I) The JC-VLP uptake in medullary vessel endothelia totally overlapped with FITC-FSA uptake (arrows). J–L) FITC-FSA uptake occurred mainly in Meca 32 negative vessels.

In kidney, BK- and JC-VLP uptake was specifically associated with microvessels of renal medulla (**Figure 7A–D**). Staining intensity of positive vessels was distinct and strong. However, VLP uptake was not seen in all medullary vessels, suggesting discrimination between different segments of vasa recta. Most of the VLP staining did not overlap with staining for Meca 32 (syn. PV-1, a component of fenestral diaphragms) (**Figure 8A–F**), which label endothelial cells of fenestrated segments of vasa recta [60], [61]. This suggests that the BK- and JC-VLP uptake took place in non-fenestrated endothelial cells, which are located in descending segments of the vasa recta. Endothelial cells that took up VLPs also rapidly and selectively endocytosed FITC-FSA (**Figure 8G–I**), which in liver is a functional marker for LSECs [30], [54], [59], [62]. FITC-FSA uptake in kidney was seen in medullary vessels only, and the uptake showed little overlap with Meca 32 staining (**Figure 8J–L**). The distribution pattern of JC-VLP and FITC-FSA in kidney medullary vessels almost completely overlapped, suggesting the presence of a highly endocytically active endothelium in these vessels.

BK- and JC-VLPs also distributed to the transitional epithelium of the renal pelvis (**Figure 7G, H**), whereas glomeruli and tubular structures of the nephron were negative in all mice (**Figure 7E, F**) except for one animal injected with BK-VLP, which showed positive staining of a few glomeruli (not shown). Lectin histochemistry for SNA and MAH showed positive labeling of all endothelia in kidney (**Figure S3**).

**Figure 9:**
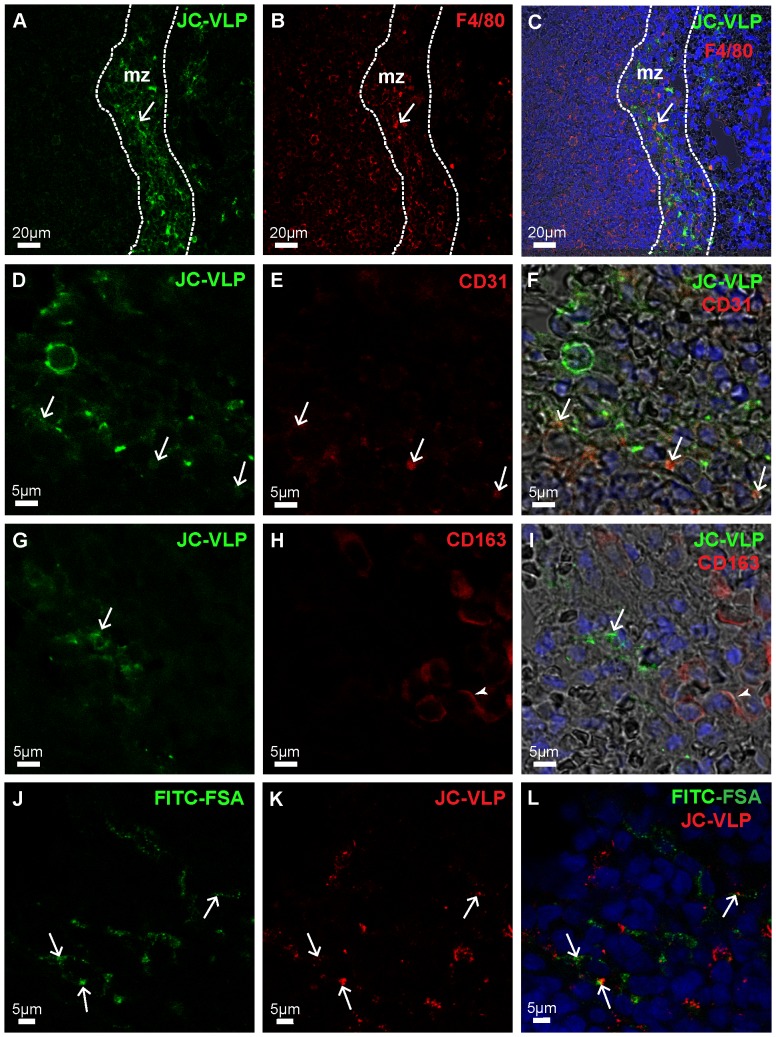
JC-VLP uptake in spleen. Panels A–I: Mouse tissues were perfusion fixed 7 min after intravenous injection of JC-VLPs. Paraffin sections of spleen were double immune labeled using a rabbit antiserum against BK-VP1 [53] (reacts also with JC-VP1), and antibodies against F4/80 (A–C), CD31 (D–F) or CD163 (G–I). Antibodies are listed in Table 1. The VP1 staining pattern (green) showed that the uptake of VLPs was concentrated in the red pulp marginal zone, mz (A), and here partly co-localized with F4/80 (C, arrow), and CD31 (F, arrows), but not with CD163 (I; arrow indicates VP1, and arrow head to CD163 staining). The F4/80 (B, C), CD31 (E, F), and CD163 (H, I) staining patterns are all shown in red. Panels J–L: In J–L, tail vein injection of JC-VLPs was followed by injection of FITC-FSA in the opposite tail vein and tissues perfusion fixed 15 min after the VLP administration. Both JC-VLP (K; red fluorescence) and FITC-FSA (J; green fluorescence) distributed to the reticuloendothelial network of the spleen red pulp marginal zone (L; arrows point to overlap of JC-VLP staining and FITC-FSA uptake).

In spleen, the BK- and JC-VLPs distributed to the marginal zone that separates the lymphocyte rich white pulp from the red pulp (**Figure 9** **, and Figure S6**). The VP1 labeling showed a net-like staining pattern in this region. Double immune labeling for VP1 and the macrophage marker F4/80 (**Figure 9A–C**), or CD31 (**Figure 9D–F**), which is expressed by many endothelial cells, revealed scattered co-localisation of VLPs with CD31 and F4/80 positive cells, suggesting that the marginal zone reticuloendothelial network of macrophages and endothelial cells are involved in VLP uptake in the spleen. Staining for the macrophage antigen CD163 [63] did not co-localize with VP1 staining (**Figure 9G–I**), indicating VLP uptake only in subsets of spleen red pulp macrophages. Some of the JC-VLP uptake in spleen co-localized with FITC-FSA which also distributed to the reticuloendothelium of the spleen red pulp marginal zone (**Figure 9J–L**).

## Discussion

Clinical models of BKV and JCV infection demonstrating replicative pathologies with close correlation to nephropathy, cystitis or multifocal leukoencephalopathy are currently lacking. However, these viruses are recognized and taken up by cells from non-permissive hosts, including mice [37], [42], allowing us to study their cellular site of uptake in a mouse model. Primary infection with these viruses is believed to occur during early childhood, but their initial infection route or organ distribution is not well known. Using well characterized VLPs, we present evidence that LSECs are highly effective in clearing a large fraction of blood-borne BKV and JCV particles. Moreover, we observed that BK and JC VLPs are efficiently taken up in some microvessels of renal medulla and the spleen marginal zone reticuloendothelium. By effectively acting as a virion sink in the absence of specific antibodies, LSECs may play a key role in the mostly asymptomatic course of primary BKV and JCV infection, leaving only little virus to escape and reach the renal medulla (and possibly the spleen), an important site of BKV and JCV persistence [40], [41]. LSECs represent a highly specialized type of scavenger endothelium preserved across different vertebrate species including rodents and humans [10], [45]. Several signature characteristics are the same in LSECs across mammals: Cell morphology and endocytosis receptor expression in human LSECs are identical to that of rodents and pig [10], [11], [15], [43], [44], and the scavenger function of rat, mouse, and pig LSECs is strikingly similar [64]. It is therefore likely that our present findings on the efficient LSEC endocytosis of polyoma VLPs in mice reflect the fate of these viruses also in human livers.

The observed biphasic pattern of polyoma VLP clearance from blood, with a rapid α-phase and a slower β-phase is comparable to the clearance kinetics of many macromolecules and colloids that are efficiently eliminated via receptor-mediated endocytosis in the LSECs [15], [25], [26]. There were, nevertheless, some interesting differences between the BK-VLP and JC-VLP clearance patterns. While JC-VLPs were almost completely removed from the circulation within 10 minutes, the α-phase was less dominant in the elimination of BK-VLPs, suggesting a rapid saturation of BK binding/uptake receptors or interaction of BK-VLPs with blood factors (proteins or cells), delaying the blood clearance. Interestingly, in their natural host cells BKV is reported to be internalized via caveolin-mediated endocytosis whereas JCV enters via clathrin-mediated endocytosis [36]. This could also be an explanation of our results, because LSECs have well-developed clathrin-mediated endocytosis machinery, and rely heavily on this type of endocytosis to maintain their signature scavenging activity [34], [65].

Our hypothesis is that LSECs protect against virus infections, due to their very efficient endocytosis and high catabolic activity which rapidly destroy internalized material [10]. Our *in vivo* data indicate that at least JC-VLPs are rapidly degraded in the liver, as reported for other macromolecules taken up by the LSECs [10], [26]. In this context, it is of interest to note that BKV viremia is readily detectable in transplant patients with BKV-associated diseases, while JCV viremia is only rarely detected in patients with JCV-associated diseases [66] [67].

However, degradation of VLPs is not a proof of cellular destruction of infective virus, and to establish whether BKV or JCV uptake in LSECs will lead to destruction of all internalized virus particles, or allow for endosomal escape and intracellular replication of a fraction of the internalized virions would require further studies. However, *in vitro* model systems are currently lacking that recapitulate the known *in vivo* physiological activity of human LSECs, hence impeding such studies.

In addition to a major hepatic uptake, a minor, but significant BK-/JC-VLP uptake was also measured in spleen red pulp and kidney medulla. The spleen red pulp is known to harbor scavenger type of endothelia [10] and could thereby serve as a secondary site of BKV and JCV clearance. Of note, the rapid and specific uptake of polyoma virus or VLPs in kidney medullary vessels has to our knowledge not been reported before, and may reveal novel insights into the infection route of BKV and JCV. It is notable, however, that clinical recommendations emphasize the importance of medullary tissue for a sensitive tissue diagnosis of BK polyomavirus-associated nephropathy in kidney transplant recipients [68]. Moreover, BKV infection of endothelial cells has been reported in a patient with AIDS and a renal transplant recipient [69], [70]. The kidney uptake reported in the present study was associated with the endothelial lining of distinct vasa recta segments. Most of the VLP-positive vessels did not express PV-1/Meca32, suggesting location of VLPs in the non-fenestrated, or descending part of vasa recta [61]. The VLP positive endothelial cells also efficiently endocytosed a functional LSEC marker and scavenger receptor ligand, FITC-FSA, suggesting high endocytic activity of these cells compared to other renal endothelia. Whether this uptake will aid in persistence or clearance of the infectious virus remains to be revealed.

The brain, which is the main site for JCV pathology (PML) [41], was not readily identified by the intravenously injected VLPs, as judged by the extremely low levels of radioactivity accumulating in this organ. This apparent lack of uptake in brain may be explained partly by the intact blood-brain and blood-cerebrospinal fluid barrier of adult mammals. However, the steps in colonization of the brain by JCV are not well characterized and it is still unclear if this occurs during primary viremia or during reactivation [41], if it requires replication competent JCV as opposed to VLPs, or access via other cell types including B-lymphocytes and/or hematopoietic progenitor cells [71]. In addition to the usually distinct VP1 amino acid mutations found [58], virus causing PML bear a rearranged non-coding control region that increases the replication potential [72].

We tested the *in vivo* fate of VLPs of the two of the most frequently identified JCV mutants, namely JC-VLP_L55F_ and JC-VLP_S269F_, both of which have lost their ability to bind alpha(2,6)-linked sialic acid residues on gangliosides, used by JCV for cellular entry [41], [58]. The rate of blood clearance was rapid, and organ distribution after 60 min was almost similar for Mad-1 and mutant JC-VLPs, suggesting that their blood clearance by scavenger cells did not depend on binding to alpha(2,6)-linked sialic acid receptors.

The high scavenging capacity of LSECs for most ligands is mediated by a limited set of high affinity endocytosis receptors [10]. Together with the mannose receptor, the scavenger receptors stabilin-1 and stabilin-2 are considered the main endocytic workhorses of these cells [10], [65], and are responsible for the uptake of a wide range of macromolecules, including oxidized LDLs, hyaluronan, chondroitin sulphate, procollagen propeptides, and advanced glycation end products [13], [30], [65]. Many serum proteins that are phosphorylated as a consequence of platelet activation and blood coagulation are also recognized by the LSEC scavenger receptors [73]. Recent data suggest that the VP1 molecules of BKV contain posttranslational modifications including phosphate groups [74]. FSA, a well-known ligand for the scavenger/stabilin receptors [30], [59] was unable to alter the clearance kinetics of blood-borne VLPs, suggesting that the LSEC scavenger receptors were not involved in the hepatic uptake of the particles. However, intracellular degradation of JC-VLPs in liver was significantly impaired. Together, these data suggest traffic of FSA and JC-VLP to the same degradation compartments, but via different receptors. However, we cannot exclude binding to different sites of the same endocytosis receptor, as non-reciprocal cross-competition is described for binding of FSA and hyaluronan to scavenger receptors/stabilin-2 in LSECs [11], [13], [25]. Mice deficient in the mannose receptor [46] showed similar clearance and organ distribution of BK/JC-VLPs as wild-type mice with normal expression of mannose receptor in LSECs. Although this is consistent with the fact that polyomavirus VLPs including BKV and JCV are not glycosylated [74], it should be noted that the mannose receptor has binding sites for non-glycosylated residues in addition to acting as a lectin [10], [75].

### Conclusions

Mouse LSECs were identified as efficient scavengers of BK- and JC-VLPs. Despite this efficient clearance mechanism, the VLPs still reached the kidney which is a key organ of BKV and JCV persistence in humans. Of note, the kidney was targeted by VLPs, i.e. in the absence of virus replication. Here we identified uptake predominantly in endothelial cells of the non-fenestrated segments of vasa recta, which also possessed high endocytic activity towards the scavenger receptor ligand FITC-FSA, a hitherto unknown function of these renal endothelial cells. A better understanding of LSEC-mediated polyomavirus clearance may shed light into the role of these cells in the development of polyomavirus-associated diseases. Moreover, this may permit the development of strategies to harness this process for immunocompromised subjects at risk of polyoma virus replication.

## Supporting Information

Figure S1 **Liver distribution of BK- and JC-VLPs – details of** **Figure 4B and 4E** **.** The figure shows the green and red channels, white field (WF) image, and merged image of immune labeled paraffin sections of liver from mice injected with BK-VLPs (A–D), or JC-VLPs (E–H). The livers were perfusion fixed 15 min after intravenous injection, and sections labeled with rabbit antiserum to BK-VP1 (cross-reacts with JC-VP1), and an antibody to the mannose receptor (MR; LSEC marker [26]). The staining patterns of VLPs (A, E; green) and MR (B, F; red) were similar (overlay in D, H), and localized exclusively to sinusoids (s; arrowheads), whereas hepatocytes (outlined in C, D, G, H) were negative. Hn, hepatocyte nucleus.
(TIFF)

Figure S2 **Uptake of JC-VLPs in LSECs in vitro – Z-stack.** Freshly isolated LSECs were incubated at 37°C for 1 h with 10 µg/ml JC-VLPs, fixed, and double immune labeled with rabbit anti-serum to BK-VP1 (red), and an antibody to an LSEC marker (the mannose receptor, MR; green). Draq5 was used to stain the cell nucleus. The panel shows the images of a Z-stack recorded by confocal laser scanning microscopy. Every image shows the same cell at different depth (Z axis) with 0.3 µm distance from the previous optical image, recorded from a given 0 µm (A) to 1.2 µm depth (E). The intensity of the Draq5 staining increased gradually from A to D, meaning that the images were taken from a layer close to the cell plasma membrane in A, towards the center of the cell in D. Positive VP1 staining (red) can be seen in all images, indicating cellular uptake of VLPs. The MR is a constitutively recycling endocytosis receptor in LSECs [14] and MR staining is strongest towards the periphery of the cell.
(TIFF)

Figure S3 **Lectin histochemistry of liver, kidney and spleen.** Panels A–H: Paraffin sections of liver (A, B), kidney medulla (C, D), kidney cortex (E, F), and spleen (G, H) were labeled with biotinylated agglutinins: *Maackia amurensis II* (MAH; A, C, E, G) that binds to alpha(2,3)-linked sialic acid, and *Sambucus nigra* (SNA; B, D, F, H) that binds to alpha(2,6)-linked sialic acid. Lectin binding to sialic acids was visualized by Alexa555-streptavidin. Both type of lectins bound to all endothelia in liver (A, B), and kidney (C–F). In spleen (G–H), the lectin staining pattern was more diffuse than in the two other organs. PV, portal vein; CV, central vein; g, glomerulus.
(TIFF)

Figure S4 **Effects of the scavenger receptor ligand FSA on uptake and intracellular degradation of JC-VLP.** Panels A–C: Rate of blood clearance, and subsequent organ distribution of ^125^I-JC-VLP in mice injected with a saturating dose (0.8 mg/mouse) of the scavenger receptor ligand FSA immediately prior to the injection of ^125^I-JC-VLP (dose: 0.5 µg/mouse). Blood samples were taken at the indicated time points in A, B and analyzed for ^125^I-labeled degradation products and intact ligand [15]. A, B) The graph in A shows decrease of intact ligand in blood as a function of time, whereas the graph in B shows increase in degradation products released into the blood with time. Results are given in cpm per µl blood. Different symbols refer to separate animals (n = 3). cpm, counts per minute. The slope of the line drawn in B represents the average release rate of degradation products in the 10–30 min period after ligand injection where this release followed approximate first order kinetics. C) Organ distribution of radioactivity 60 min after the intravenous administration of ^125^I-JC-VLP and FSA. *Significantly more radioactivity (p-value <0.01) was recovered in liver after 60 min following competitive inhibition with FSA (Figure S4-C; 44%), than after injection with ^125^I-JC-VLP alone (Figure 3B; 30%). U bladder, urine bladder; GI tract, gastrointestinal tract; FSA, formaldehyde denatured serum albumin.
(EPS)

Figure S5 **Blood clearance and tissue distribution of ^125^I-JC-VLPs in MR^-/-^ mice.** C57BL/6 MR^-/-^ mice (n = 2) were injected intravenously with approximately 0.5 µg of ^125^I-JC-VLP, and blood samples taken at the time points indicated in A and B and analyzed for ^125^I-labeled degradation products and intact ligand [15]. After 60 min, the animals were euthanized, then perfused free of blood, and radioactivity measured in tissues and organs. A) Kinetics of decrease of intact ^125^I-labeled ligand in blood. B) Increase in ^125^I-degradation products released into the blood with time. Results in A and B are given in counts per minute (cpm) per µl blood. C) Organ distribution of radioactivity 60 min after injection of ligand. Recovered radioactivity in organs and tissues at this time point was taken as 100%.
(EPS)

Figure S6 **Uptake of blood-borne BK-VLPs in spleen.** The figure shows the distribution of BK-VLPs in spleen 15 min after intravenous injection. Paraffin sections were stained with anti-BK-VP1 (Table 1) and Alexa488-goat-anti-rabbit antibody (green fluorescence). The VLPs were taken up in the reticuloendothelial network in the spleen red pulp marginal zone (mz, arrowheads). rp, red pulp; wp, white pulp.
(TIF)
