# Supplementary figures and images for: Efficient Uptake of Blood-Borne BK and JC Polyomavirus-Like Particles in Endothelial Cells of Liver Sinusoids and Renal Vasa Recta
Source: PLoS One. 2014 Nov 6;9(11):e111762. doi: 10.1371/journal.pone.0111762 (PMC4222947; doi:10.1371/journal.pone.0111762)

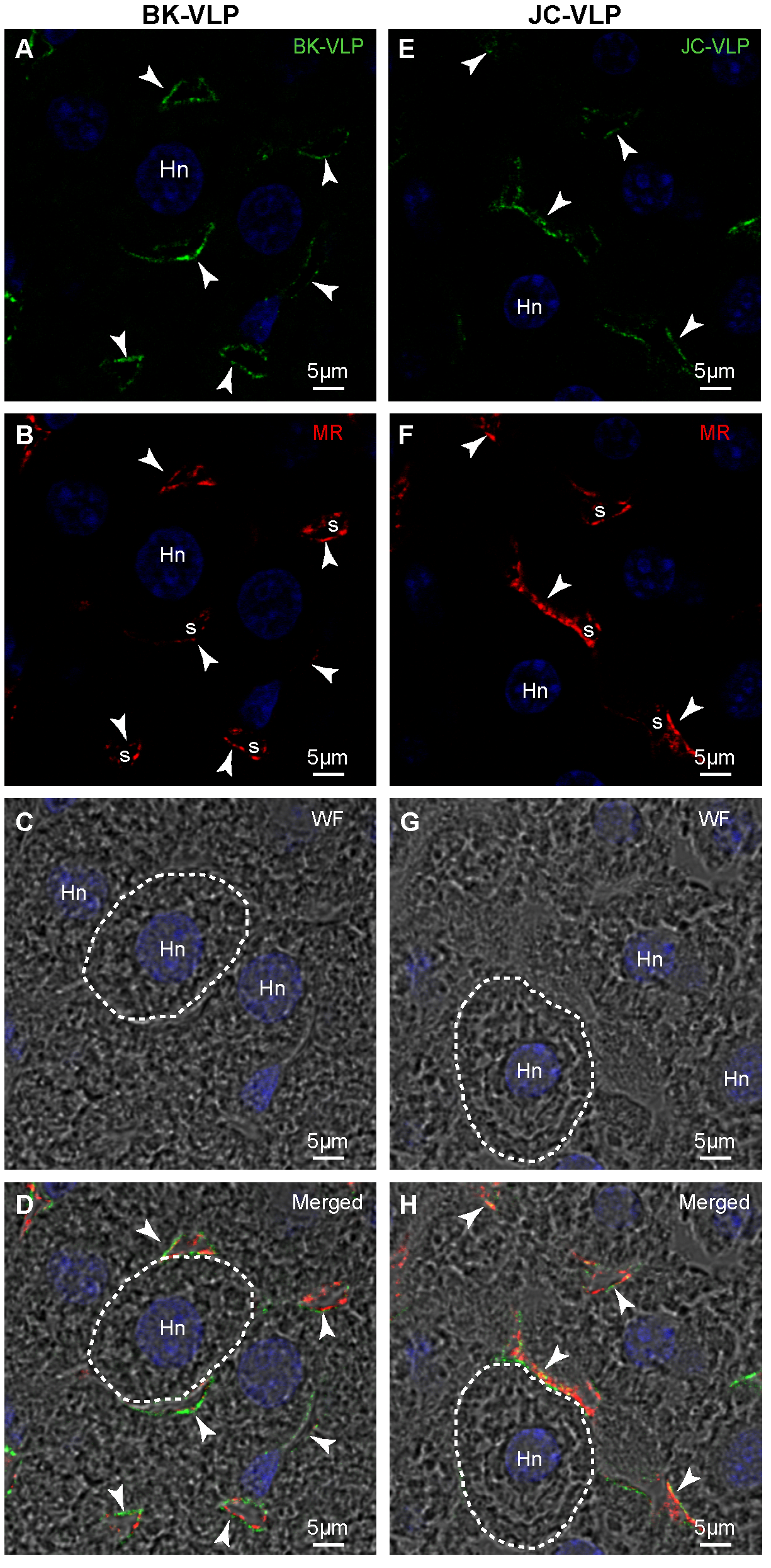

Supplement: Figure S1 — Liver distribution of BK- and JC-VLPs – details of Figure 4B and 4E . The figure shows the green and red channels, white field (WF) image, and merged image of immune labeled paraffin sections of liver from mice injected with BK-VLPs (A–D), or JC-VLPs (E–H). The livers were perfusion fixed 15 min after intravenous injection, and sections labeled with rabbit antiserum to BK-VP1 (cross-reacts with JC-VP1), and an antibody to the mannose receptor (MR; LSEC marker [26]). The staining patterns of VLPs (A, E; green) and MR (B, F; red) were similar (overlay in D, H), and localized exclusively to sinusoids (s; arrowheads), whereas hepatocytes (outlined in C, D, G, H) were negative. Hn, hepatocyte nucleus. (TIFF) [file pone.0111762.s001.tiff]

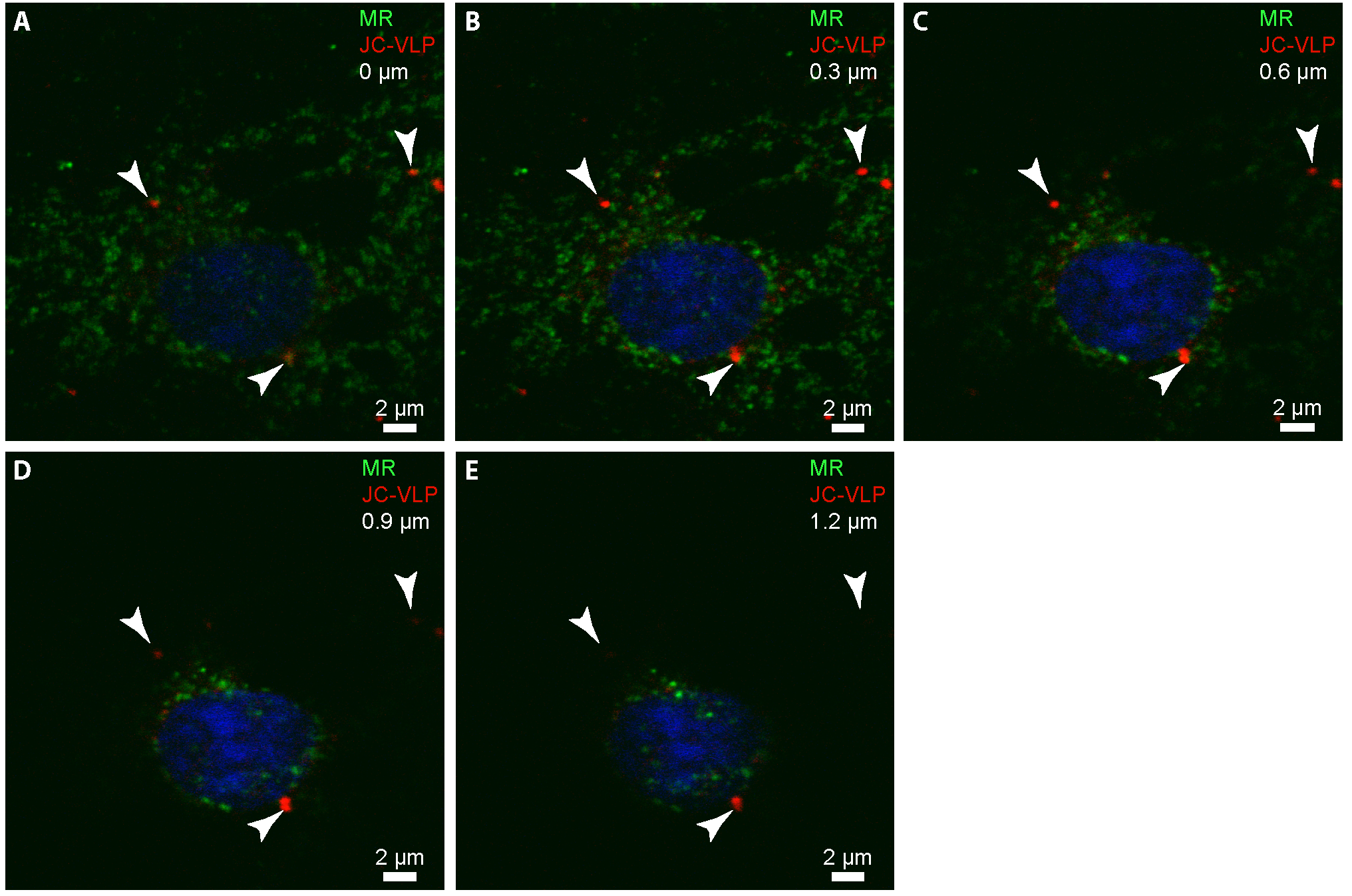

Supplement: Figure S2 — Uptake of JC-VLPs in LSECs in vitro – Z-stack. Freshly isolated LSECs were incubated at 37°C for 1 h with 10 µg/ml JC-VLPs, fixed, and double immune labeled with rabbit anti-serum to BK-VP1 (red), and an antibody to an LSEC marker (the mannose receptor, MR; green). Draq5 was used to stain the cell nucleus. The panel shows the images of a Z-stack recorded by confocal laser scanning microscopy. Every image shows the same cell at different depth (Z axis) with 0.3 µm distance from the previous optical image, recorded from a given 0 µm (A) to 1.2 µm depth (E). The intensity of the Draq5 staining increased gradually from A to D, meaning that the images were taken from a layer close to the cell plasma membrane in A, towards the center of the cell in D. Positive VP1 staining (red) can be seen in all images, indicating cellular uptake of VLPs. The MR is a constitutively recycling endocytosis receptor in LSECs [14] and MR staining is strongest towards the periphery of the cell. (TIFF) [file pone.0111762.s002.tiff]

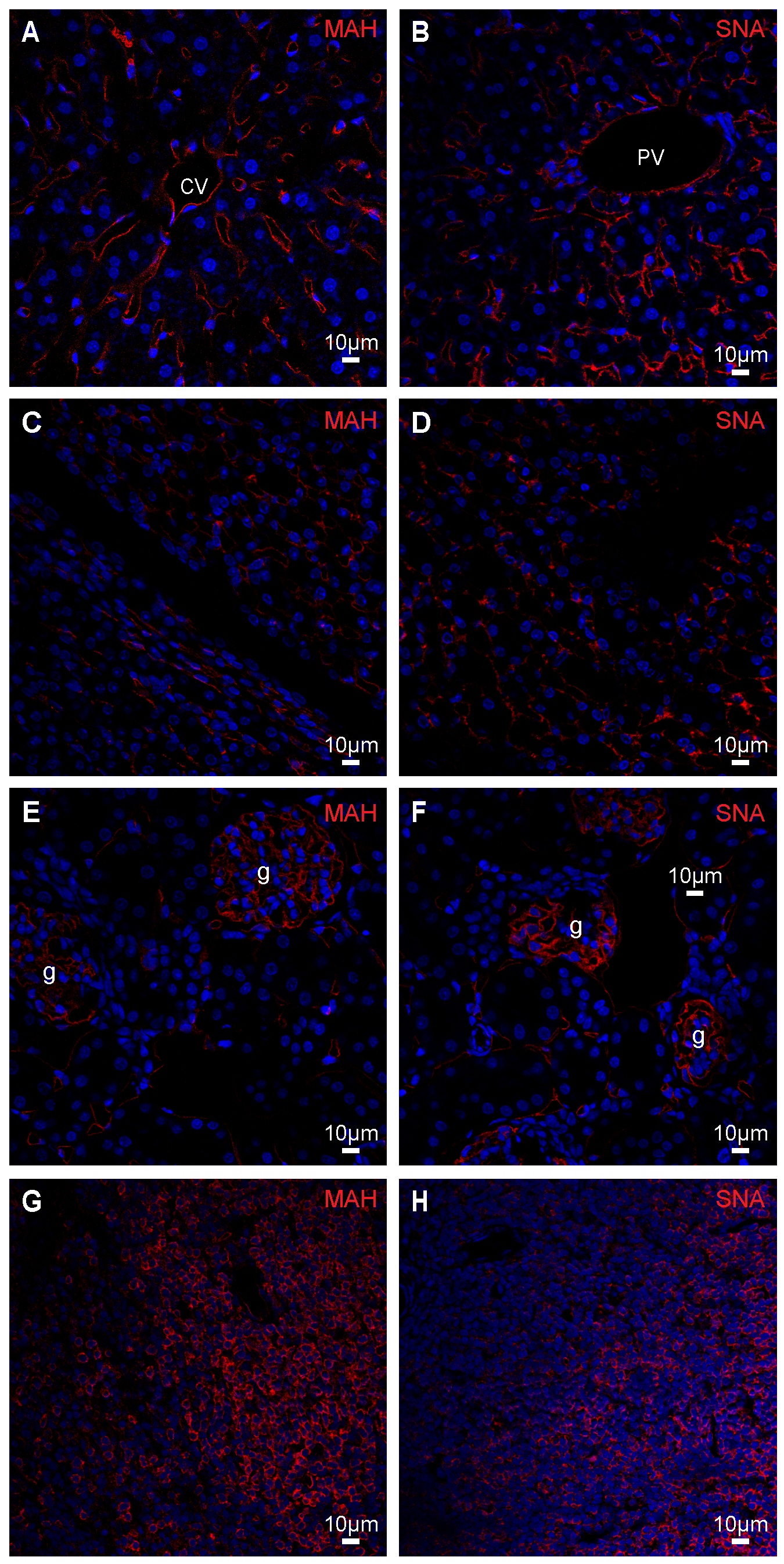

Supplement: Figure S3 — Lectin histochemistry of liver, kidney and spleen. Panels A–H: Paraffin sections of liver (A, B), kidney medulla (C, D), kidney cortex (E, F), and spleen (G, H) were labeled with biotinylated agglutinins: Maackia amurensis II (MAH; A, C, E, G) that binds to alpha(2,3)-linked sialic acid, and Sambucus nigra (SNA; B, D, F, H) that binds to alpha(2,6)-linked sialic acid. Lectin binding to sialic acids was visualized by Alexa555-streptavidin. Both type of lectins bound to all endothelia in liver (A, B), and kidney (C–F). In spleen (G–H), the lectin staining pattern was more diffuse than in the two other organs. PV, portal vein; CV, central vein; g, glomerulus. (TIFF) [file pone.0111762.s003.tiff]

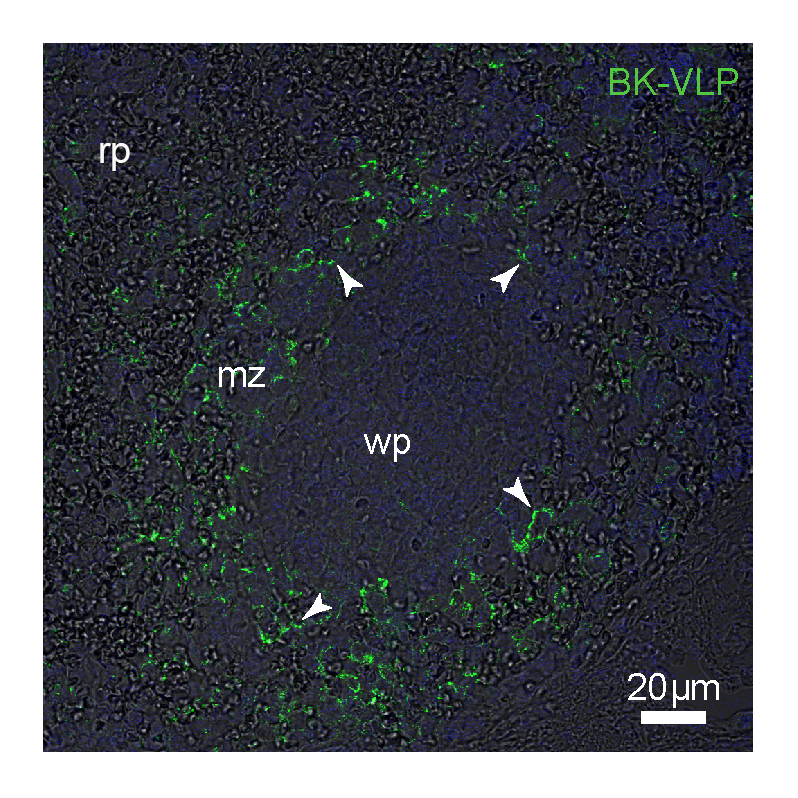

Supplement: Figure S6 — Uptake of blood-borne BK-VLPs in spleen. The figure shows the distribution of BK-VLPs in spleen 15 min after intravenous injection. Paraffin sections were stained with anti-BK-VP1 (Table 1) and Alexa488-goat-anti-rabbit antibody (green fluorescence). The VLPs were taken up in the reticuloendothelial network in the spleen red pulp marginal zone (mz, arrowheads). rp, red pulp; wp, white pulp. (TIF) [file pone.0111762.s006.tif]
